# Guidelines on Vaccinations in Paediatric Haematology and Oncology Patients

**DOI:** 10.1155/2014/707691

**Published:** 2014-04-29

**Authors:** Simone Cesaro, Mareva Giacchino, Francesca Fioredda, Angelica Barone, Laura Battisti, Stefania Bezzio, Stefano Frenos, Raffaella De Santis, Susanna Livadiotti, Serena Marinello, Andrea Giulio Zanazzo, Désirée Caselli

**Affiliations:** ^1^Paediatric Hematology Oncology, Azienda Ospedaliera Universitaria Integrata Ospedale Borgo Roma, P.le L.A. Scuro 10, 37134 Verona, Italy; ^2^Paediatric Hematology Oncology, Regina Margherita Hospital, P.zza Polonia 94, 10126 Torino, Italy; ^3^Paediatric Hematology, G. Gaslini Institute, Via Gerolamo Gaslini 5, 16148 Genova, Italy; ^4^Paediatric Hematology Oncology, Azienda Ospedaliera, Via Gramsci 14, 43100 Parma, Italy; ^5^Paediatrics, Azienda Ospedaliera, Via Lorenz Böhler 5, 39100 Bolzano, Italy; ^6^Paediatric Hematology Oncology, Azienda Ospedaliera Universitaria Meyer, Viale Pieraccini 24, 50139 Firenze, Italy; ^7^Paediatric Hematology Oncology, Casa Sollievo della Sofferenza Hospital, Viale Cappuccini 2, 71013 San Giovanni Rotondo, Italy; ^8^Paediatric Immunology and Infectious Diseases, Ospedale Bambin Gesù, Piazza di Sant'Onofrio, 4, 00165 Roma, Italy; ^9^Infectious Diseases, Azienda Ospedaliera, Via Giustiniani, 35128 Padova, Italy; ^10^Paediatric Hematology Oncology, Burlo Garofalo Institute, Via dell'Istria 65, 34137 Trieste, Italy; ^11^Medical Direction, A.O.U. Meyer, Children Hospital, Viale Pieraccini, 24, 50139 Firenze, Italy

## Abstract

*Objective.* Vaccinations are the most important tool to prevent infectious diseases. Chemotherapy-induced immune depression may impact the efficacy of vaccinations in children. *Patients and Methods*. A panel of experts of the supportive care working group of the Italian Association Paediatric Haematology Oncology (AIEOP) addressed this issue by guidelines on vaccinations in paediatric cancer patients. The literature published between 1980 and 2013 was reviewed. *Results and Conclusion*. During intensive chemotherapy, vaccination turned out to be effective for hepatitis A and B, whilst vaccinations with toxoid, protein subunits, or bacterial antigens should be postponed to the less intensive phases, to achieve an adequate immune response. Apart from varicella, the administration of live-attenuated-virus vaccines is not recommended during this phase. Family members should remain on recommended vaccination schedules, including toxoid, inactivated vaccine (also poliomyelitis), and live-attenuated vaccines (varicella, measles, mumps, and rubella). By the time of completion of chemotherapy, insufficient serum antibody levels for vaccine-preventable diseases have been reported, while immunological memory appears to be preserved. Once immunological recovery is completed, usually after 6 months, response to booster or vaccination is generally good and allows patients to be protected and also to contribute to herd immunity.

## 1. Introduction

In the last century, paediatric morbidity and mortality due to common infectious diseases were impressively reduced in developed countries through the introduction of vaccination practices [1–3]. Nowadays, vaccination programs represent a universally recognized tool to prevent the spread of many infectious agents and to reduce death and disability worldwide. For some vaccine-preventable diseases, definitive eradication has thus been achieved, such as smallpox worldwide and poliomyelitis throughout the USA and Europe [3, 4]. Vaccination plans have been continuously updated during the last two decades due to either availability of new vaccines, such as for herpes varicella-zoster virus (VZV), pneumococcus and meningococcus C, and human papilloma virus (HPV), or the identification of new groups and age ranges in whom the use of vaccination results in an improvement of health [5].

Paediatric malignant diseases today represent the second major cause of death in developed countries. Survival rates have improved over the last 3 decades due to the use of a multidisciplinary approach based on chemotherapy, surgery, radiotherapy, and hematopoietic stem cell transplantation and to improved supportive therapies [6].

One major draw-back of chemotherapy is the immune suppression of the patient, that requires up to 6–12 months after the end of treatment to recover [7–14]. This may have a negative impact on the efficacy of vaccinations because of complete or partial loss of protective serum antibody titres, unavoidably reduced compliance to scheduled vaccination, and the coexistence of other defects of the immune system, e.g., functional asplenia [15]. Generally, the patients cured of paediatric malignancies are not considered at higher risk of serious infectious complications than the healthy population, except for those who have been splenectomised or with a persistence of a functional asplenia. Yet, limited reports are available on long-term risk of severe infection; a persistent defect of humoral response to infection by* Haemophilus influenzae* or to vaccination for* Streptococcus pneumoniae*, measles, and rubella has been reported [11, 16]. Thus, the real need for vaccinations during and after treatment for paediatric cancer remains a relevant issue. This topic was addressed in part in previous recommendations for immune-compromised host published in 1993 and 2002; yet, the resulting statements for cancer patients were different for vaccination both during and after chemotherapy [17].

The aim of this paper is to analyse and discuss among experts, following an accepted methodology, the available literature in order to propose updated guidelines for the vaccination of paediatric cancer patients. This document does not extend to patients who have undergone hematopoietic stem cell transplant because specific guidelines have already been provided by leading scientific societies [18].

## 2. Methods 

This project was developed according to the methodology suggested by the infectious diseases society of America (IDSA) [19]. A panel of experts from the working group for supportive care of the Italian Association Paediatric Hematology Oncology (AIEOP) jointly defined the questions to be answered:

- is there sufficient evidence to support vaccination during chemotherapy? If so, who should be vaccinated?
- what is the evidence concerning vaccination after completion of chemotherapy, and when should vaccinations be scheduled?
- are vaccinations indicated for parents and other family members? If so, who should be vaccinated and when?

The panel of experts was divided into subgroups to separately review the literature on different topics: diphtheria, tetanus, pertussis (F.F., M.G., A.B.), poliomyelitis, influenza, HPV (M.G., S.B., and F.F.), hepatitis B (A.B., S.C., and F.F.), hepatitis A (L.B., S.C., and M.G.), measles, rubella, varicella (S.C., S.M, and G.Z.), meningococcus,* Haemophilus*, and pneumococcus (S.F., S.L., R.D.S., and D.C.).

Relevant articles in English language were selected by literature search on Pub Med (http://www.ncbi.nlm.nih.gov/PubMed/) using key-words as vaccination, pediatric, and chemotherapy. The time frame of the literature search was from January 1st, 1980 to June 30th, 2013. References of papers selected by the literature search were further assessed to identify additional relevant papers. Strength of recommendation and quality of evidence were scored as reported in Table 1.

The conclusions achieved by individual groups were presented in 2 plenary discussions before final approval. In case of disagreement, the issue was settled by discussion with an external expert. The final draft was reviewed by a second external reviewer.

## 3. Results and Discussion

### 3.1. Vaccination of Patients during Chemotherapy

The nonlive vaccines based on toxoid, protein subunits, bacterial antigens, or immunogenic proteins obtained with recombinant technology are not contraindicated in principle during chemotherapy [20, 21]. This category includes vaccines for tetanus, diphtheria, pertussis, poliomyelitis, hepatitis B, influenza,* Haemophilus*, pneumococcus, and meningococcus. Although human papilloma virus (HPV) vaccine belongs to this group, no indication can be formulated by the panel of experts at present due to insufficient knowledge [22].

The major drawback of performing these vaccinations during the chemotherapy program is the potentially suboptimal antibody response, resulting in reduced efficacy compared to a healthy child. Yet, to follow the recommended schedule of immunisation, this may be done provided the patient is in good clinical conditions and not affected, or at specific risk to be affected, by infection or significant organ toxicity for at least 3 weeks after vaccination.

For poliomyelitis, tetanus, diphtheria, and pertussis, limited data are available on vaccination during chemotherapy; [23–26] thus, the level of evidence was scored as optional (C, III). These vaccinations are compulsory or highly-recommended worldwide and most patients are expected to have completed (3 doses) or almost completed (2 doses) the primary vaccination schedule before paediatric malignancy is diagnosed. The need for maintaining during chemotherapy a protective level of serum antibodies for poliomyelitis and diphtheria is in part attenuated by the protection afforded by herd immunity, given the high percentage of protective antibody titres present in the healthy population.

For tetanus, policies for the management of the at-risk wound, such as washing of the wound, the use of antibiotics, and passive immunoprophylaxis, are effective preventive measures that reduce the need for active immunization while the patient is on chemotherapy.

For pertussis, active immunization has been shown to be feasible in HIV patients, although the response was lower than in children not immune-compromised; no reports are available for paediatric cancer patients [25].

Several authors have assessed the efficacy of vaccination for hepatitis B virus (HBV) and hepatitis A virus (HAV) early after the diagnosis of paediatric malignancy [27–35]. This measure is generally adopted in countries with high prevalence of HBV or HAV infection, in which vaccination is not compulsory due to limited health resources. These studies showed that vaccination of seronegative patients for HBV and HAV in the early phase of chemotherapy reduces the risk of contracting hepatitis and confers protection to immune-compromised patients, although at a lower rate than in healthy populations or in patients off-therapy. The level of evidence was scored as moderately recommended for HBV (B, II). For HAV vaccination, the level of evidence was scored as optional (C, III) since passive immune-prophylaxis is equally effective in preventing acute hepatitis. However, the vaccination for HAV is recommended in seronegative patients with preexisting cirrhosis or other hepatic diseases or in patients living in or travelling to highly endemic countries for HAV.

Influenza is one of the community-acquired respiratory infections that can cause a significant morbidity in paediatric oncology patients, due to frequent complication by bacteremia [36] requiring hospitalization, which can be prevented. by vaccine. Several authors have shown that vaccination for influenza may generate immune responses also in children receiving chemotherapy, although at lower rates than in healthy children or children off-chemotherapy. Most of the studies included patients during the less-intensive phases of treatment, such as the maintenance phase for acute lymphoblastic leukemia [37–53]. A fourfold rise of the protective antibody serum level was found in 25% to 52% of patients [45]. Despite that, data on the impact of vaccination on the clinical outcome and seasonal morbidity for these patients are still lacking [50]. In patients receiving current chemotherapy for high-risk leukaemia, a profound reduction of B-cell lymphocyte number and function has been reported, potentially affecting immune response to influenza vaccination during the maintenance phase [51]. Overall, the level of evidence for influenza was moderately recommended (B, II).

Live attenuated influenza vaccine is not indicated in the immune-compromised host and only the inactivated preparation is allowed [54].

Invasive infections by capsulated bacteria may represent severe complications during chemotherapy, especially in leukaemic patients in whom an impairment of pneumococcal immunity has been reported [15, 16]. There is limited experience on the use of vaccinations for Pneumococcus, Haemophilus, and Meningococcus [55–61]; in one case, a failure is reported [62]. Effective prevention remains based on prompt antibiotic treatment of febrile at-risk patients and isolation measures to prevent contact especially during periods of severe neutropenia. The level of evidence was scored as optional (C, III).

The use of attenuated-virus vaccination for measles-mumps-rubella is usually not indicated for patients on chemotherapy because they are at higher risk of fever or vaccine disease by vaccine strain [63]. In a very small cohort of patients with leukaemia vaccinated for measles (8 patients) and mumps (4 patients), transient withdrawal of chemotherapy was associated with a better seroconversion rate and tolerability. Yet, no significant conclusions can be drawn for current practice [64]. Thus, the use of these vaccinations was scored as D, III. In case of measles epidemic, considering the high morbidity and the potential for mortality in immune-compromised patients, the panel of experts suggests that the risk/benefit ratio of vaccination is individually assessed for each patient; as in HIV patients, evidence of an adequate CD4+ count may assist in the decision [64]. In this context, vaccination for measles has been scored as optional (C, III).

Luthy et al. reviewed 7 studies performed over the last 3 decades to evaluate the safety and efficacy of live-attenuated VZV vaccine administered to children with acute lymphoblastic leukaemia during maintenance therapy [63]. Overall, vaccination for VZV resulted in effective seroprotection with no impact on the risk of leukaemia relapse compared to unimmunized controls [65]. The rate of failure to protect from varicella was 10–13% and the development of herpes zoster was 1–3%. The major drawback of this choice is the need for withdrawal of chemotherapy for 2 weeks, the occurrence of a vaccine disease in up to 20%–50% of the patients with the consequent need for their isolation, and the potential risk of developing a varicella-like illness [64, 66–73]. A lymphocyte count >0.7–1.0 × 10^9^/L and a platelet count >100 × 10^9^/L in patients in remission for at least 12 months are considered safe and effective to vaccinate leukemic patients for VZV [72, 73]. Vaccination is not recommended during profound leukopenia (neutrophils <0.5 × 10^9^/L, lymphocytes <0.7 × 10^9^/L) or during full-dose steroid therapy (>7 days with ≥2 mg/kg/day of prednisone or ≥0.4 mg/kg/day of dexamethasone) alone or combined with other immunosuppressive drugs. VZV vaccination during induction chemotherapy for acute leukaemia remains associated with the risk to cause fatal, disseminated disease by the live-attenuated strain due to the heavy immunosuppression of the patients [74, 75].

The risk of mortality of varicella significantly decreased over the last 20 years with the introduction of acyclovir and, more recently, of other effective agents such as foscarnet and cidofovir [76, 77]. Taken altogether, the potential side effects must be weighed against the real benefits in any decision to vaccinate for HVZ seronegative leukemic patients while they are on therapy [76–78]. Given the lower risk of mortality for patients on maintenance therapy for acute lymphoblastic leukaemia (ALL), and the availability of effective antiviral drugs, the expert panel suggests that postponing vaccination for VZV until after completion of chemotherapy is an equally safe option. For these reasons, the recommendations of the experts for VZV vaccination was C, II.

Table 2 summarizes the levels of evidence for each vaccination of patients during chemotherapy.

In summary, vaccination of patients during chemotherapy, except for HBV and HAV, is recommended only during the phase of lower intensity of treatment, as indicated by a lymphocyte count > 1.0 × 10^9^/L, thus allowing the patient to mount an adequate immune response and/or to reduce the risks of side effects. Vaccinations that have the most benefit for patients are those for HBV and Influenza. Apart from VZV during maintenance for ALL patients, the administration of live-attenuated-virus vaccines is not indicated during chemotherapy.

### 3.2. Vaccination for the Family Members of Patients on Chemotherapy

During chemotherapy, vaccinations are not contraindicated in the patient's family members. The advice is to adhere to the schedule recommended by health plan or, if seronegative, to be vaccinated both for inactivated and live-attenuated vaccines (varicella, measles, mumps, and rubella). Also in the family members the inactivated poliovirus vaccine is recommended instead of oral polio [77]. Given that influenza is a highly seasonal contagious disease with higher morbidity and severe complications in cancer patients [36], it is recommended to vaccinate for influenza all family members during the fall season before the start of the epidemic period [79, 80]. This measure contributes significantly to protecting the patient and reducing the risk of influenza. Considering the risk of VZV for an immune-compromised patient, vaccination for VZV is recommended for any family member who has no history of previous varicella (or is seronegative) [81]. The risk of transmission of virus strain vaccine by vaccinated family members is documented only in case of occurrence of postvaccination rash [77]. In this case, the temporary removal of this family member is recommended.

### 3.3. Vaccination after the End of Chemotherapy

Most authors found that chemotherapy is associated with the disappearance of vaccination immunity in patients who had completed the vaccination schedule before starting chemotherapy [82–93]. The incidence of lack of protective antibody titres measured 6–12 months after chemotherapy varied according the type of vaccine: it was higher for HBV (about 50% of patients) whilst it was lower for measles, mumps, rubella (between 20% and 40%), and polio-diphtheria-tetanus (between 10% and 30%) [12, 25, 65, 76, 82–86]. Although there is no clear correlation between the wide variation in the preservation of vaccine immunity and the type of cancer, i.e., lymphoid versus myeloid versus solid tumour, the intensity of chemotherapy regimen has been advocated by Ek et al. to explain the insufficient immune response to tetanus, diphtheria, and* Haemophilus influenzae b* vaccination after chemotherapy for high-risk acute lymphoblastic leukaemia, because of a delayed immune recovery and a low number of memory B cells [14, 94]. A recent study of immune reconstitution showed that the recovery of newly developed transitional B cells and naïve B and T cells occurs rapidly, within months, whereas the recovery of memory B and T cells is slower and can be incomplete up to 5 years. In contrast, plasmablast B cells were not affected by chemotherapy and were higher than normal in the first months of follow-up. Moreover, immunoglobulin levels normalized within weeks from the end of chemotherapy and, importantly, functional T responses to antigens such as* Cytomegalovirus*,* Herpes simplex 1*, VZV,* Candida, Tetanus*, and* Diphtheria* were normal either within or after one year from the end of chemotherapy. These findings would explain the reported good responses to booster administration despite a long-lasting deficit of B and T memory cells [85].

Most authors agree that the interval time of 6–12 months is adequate to achieve a sufficient immune recovery that, in turn, has a key role in determining the response to vaccination [14, 20, 83, 85–88, 91, 95, 96], but some studies have shown good results after reimmunization with inactivated vaccines already at 3 months [87].

The score assigned to revaccination or booster administration for poliomyelitis, tetanus, diphtheria, pertussis, HBV, measles, mumps, rubella, meningococcus, Haemophilus influenza, pneumococcus, influenza, and VZV is B, II. Table 2 summarizes the scores assigned for vaccinations after chemotherapy.

Different from other vaccinations, influenza vaccination is recommended as early as 3 months after the end of chemotherapy to confer protection while the patient is still at risk of more severe infection and complication [10, 46]. For HAV and HPV, the recommendations are based mainly on the opinion of experts because no specific data have so far been produced in these settings [22, 28] (C, III).

Considering the frequency of the loss of protective serum antibody levels after chemotherapy and the high rate of seroconversion reported with a booster or revaccination, it is not considered mandatory to measure antibody titres to decide the revaccination as well as routine checking of antibody titre response after vaccination (B, II).

In patients who stopped the course of the vaccination schedule during chemotherapy, the indication is to resume the program starting from the suspended dose (C, III).

In conclusion, chemotherapy results in a reduction of serum antibody levels for vaccine-preventable disease while immunological memory seems to be preserved. Once immunological recovery is complete, the response to vaccination is generally good, allowing patients to be protected and to contribute to herd immunity.

## Figures and Tables

**Table 1:** Scoring system used for the recommendations.

Strength of recommendation	Quality of evidence
A: strong evidence for efficacy and substantial clinical benefit; strongly recommended	I: evidence from at least one well-executed randomized, controlled trial
B: strong or moderate evidence for efficacy, but only limited clinical benefit; generally recommended	II: evidence from at least one well-designed clinical trial without randomization; cohort or case-controlled analytic studies (preferable more than one centre), from multiple time-series studies; dramatic results of uncontrolled experiments
C: insufficient evidence for efficacy or efficacy does not outweigh possible adverse consequences; optional	III: evidence from opinions of respected authorities based on clinical experience, descriptive studies, or reports of expert committees
D: Moderate evidence against efficacy or for adverse outcome; generally not recommended	
E: strong evidence against efficacy or for adverse outcome; never recommended	

**Table 2:** Summary of level of evidence and recommendations for paediatric patients during and after chemotherapy.

Vaccine	During chemotherapy		After chemotherapy	
	Level of evidence, Reference	Concise recommendation	Level of evidence and reference	Concise recommendation
Poliomyelitis	C III [21, 23]	Benefit of herd immunity Postpone if lymphocyte count <1.0 × 10^9^/L**	B II, [10, 11, 57, 85, 87–89, 91]	Booster or vaccination 6 months after stopping chemotherapy

Diphteria	C III [21, 24]	As above, passive immunoprophylaxis and antibiotic prophylaxis in case of epidemic	B II [10, 11, 14, 26, 61, 86–91]	Booster or vaccination 6 months after stopping chemotherapy (adult type vaccine for age >6 years)

Tetanus	C III [21, 24]	Postpone if lymphocyte count <1.0 × 10^9^/L** Passive immunoprophylaxis, thorough washing and disinfection of wound, and antibiotic therapy for wounds at risk	B II [11, 14, 26, 56, 61, 86, 87, 89, 91–93]	Booster or vaccination 6 months after stopping chemotherapy

Pertussis	C III [21, 25]	Postpone if lymphocyte count <1.0 × 10^9^/L** Passive immunoprophylaxis and antibiotic prophylaxis in case of epidemic	B II [10, 26, 86, 87, 90, 92]	Booster or vaccination 6 months after stopping chemotherapy

Hepatitis A virus	C III [21, 27–30]	Vaccination of the seronegative patients before starting chemotherapy in highly endemic areas; alternatively, passive immuneprophylaxis	C III [29]	Booster or vaccination 6 months after stopping chemotherapy

Hepatitis B virus*	B II [21, 28, 31, 32, 34, 35]	As above	B II	Booster or vaccination 6 months after stopping chemotherapy

Influenza	B II [21, 36–51, 53]	Vaccination yearly during fall; postpone if lymphocyte count <1.0 × 10^9^/L;** Vaccination of family members Not administered to infants <6 months of age	B II [14, 46, 49, 51, 52]	Fall Season vaccination after 3 months from stopping intensive chemotherapy Not administered to infants <6 months of age

Meningococcus^§^	C III [21, 56, 57]	Recommended vaccination prior to splenectomy Postpone vaccination if lymphocyte count <1.0 × 10^9^/L** Not administered if age <2 years	B II [87]	Not administered if age <2 years Booster or vaccination 6 months after stopping chemotherapy Booster after 3 years if vaccinated at age of 2–6 years

Haemophilus influenzae	C III [21, 24, 58, 61]	Not administered if age <2 months Recommended vaccination prior to splenectomy Postpone vaccination if lymphocyte count <1.0 × 10^9^/L°°	B II [11, 14, 87, 89]	Not administered if age <2 months Booster or vaccination 6 months after stopping chemotherapy

Pneumococcus^§,§§^	C II [20, 41, 55–58]	Recommended vaccination prior to splenectomy Postpone vaccination if lymphocyte count <1.0 × 10^9^/L**	B II [87]	Booster or vaccination 6 months after stopping chemotherapy

Measles, Mumps, Rubella	D III [21, 64]	Not administered if age <12 months Passive immunoprophylaxis in case of contact Vaccination of seronegative family members	B II [11, 12, 56, 61, 86, 87, 89, 92]	Not administered if age <12 months Booster or vaccination 6 months after stopping chemotherapy
Measles	CIII (if epidemics) [64]	In case of epidemic, patient vaccination if adequate CD4+ immune recovery°		

Varicella	C II [21, 63, 67, 69–73]	Postpone if lymphocyte count <0.7–1.2 × 10^9^/L** or the patient is not in remission for 12 months or is doing radiotherapy Not administered if age <12 months Vaccination of family members at risk; Postexposure prophylaxis within 96 hours from contact: hyperimmune Ig (0.2 mL/kg, max 10 mL) 96 hours after contact Acyclovir 4 × 20 mg/kg/day from the 7th to 21st days**	B II [11, 57, 65, 89]	Not administered if age <12 months Booster or vaccination 6 months after stopping chemotherapy

Human papilloma virus	No data		C III [22]	Not administered if age <9 years Booster or vaccination 6 months after stopping chemotherapy

Rotavirus	No data		No data	

Legend:

*Observe a 4-week interval between 1st and 2nd doses and 3-month interval between the 3rd and 4th doses of vaccine for hepatitis B virus.

^§^Meningococcal and pneumococcal polysaccharide vaccines are not effective in children <2 years.

^§§^Minimum age for conjugated vaccine is 6 weeks of age. Use pneumococcal conjugate vaccine followed, after at least 2 months, by the 23 polysaccharide vaccine. In case of splenectomy, give a booster after surgery.

°Threshold level of CD4+ recovery for MMR vaccination: CD4+ > 0.75 × 10^9^/L for children <12 months; CD4+ > 0.5 × 10^9^/L for children aged 1–5 years; >0.2 × 10^9^/L for children >6 years old and adults.

**As suggested by [72, 73].

°°Expert panel opinion. The use of acyclovir as postexposure prophylaxis has been successfully reported in immunocompetent host contacts with VZV.
